# Hemostasis Using Esophageal Balloon of Sengstaken–Blakemore Tube for Ulcer Bleeding at Esophagogastric Anastomosis: A Case Report

**DOI:** 10.3390/reports8040241

**Published:** 2025-11-20

**Authors:** Jonghoon Yoo, Taekwon Kim

**Affiliations:** Department of Emergency Medicine, Dongsan Medical Center, Keimyung University School of Medicine, Daegu 42601, Republic of Korea

**Keywords:** upper gastrointestinal bleeding, Sengstaken–Blakemore tube, hemostasis, emergency endoscopy

## Abstract

**Background and Clinical Significance**: Sengstaken–Blakemore tube insertion is a temporary but important intervention for uncontrolled upper gastrointestinal bleeding, especially when endoscopic hemostasis fails. **Case presentation**: We present the case of a 63-year-old man with a history of esophageal cancer surgery and gastric variceal treatment who presented to the emergency department with hematemesis and altered consciousness. Endoscopy revealed a bleeding ulcer at the intrathoracic esophagus. Endoscopic band ligation failed, and the patient’s condition deteriorated, prompting the insertion of an Sengstaken–Blakemore tube. Owing to prior Ivor Lewis surgery, the gastric balloon was not used; only the esophageal balloon was inflated, and hemostasis was successfully achieved. Despite the relative contraindication of prior esophageal surgery, no complications occurred. The patient was discharged on hospital day 20 without recurrence. **Conclusions**: This case illustrates that in patients with unstable upper gastrointestinal bleeding with surgical history, selective use of Sengstaken–Blakemore tube may offer life-saving hemostasis when endoscopy fails, even when standard indications are not met.

## 1. Introduction and Clinical Significance

Upper gastrointestinal bleeding remains a significant medical emergency with considerable morbidity and mortality, affecting 50–150 per 100,000 adults annually [1,2]. While variceal bleeding accounts for approximately 5–30% of all upper gastrointestinal bleeding cases [3,4], non-variceal causes, including peptic ulcers, Mallory–Weiss tears, and anastomotic ulcers, represent the majority of cases [5,6]. The mortality rate for acute upper gastrointestinal bleeding ranges from 2 to 10% in non-variceal bleeding and can reach 20–30% in variceal hemorrhage, particularly in patients with underlying liver cirrhosis [7].

Endoscopic hemostasis has become the cornerstone of initial management for upper gastrointestinal bleeding, with success rates exceeding 90% in experienced centers [8,9]. However, endoscopic treatment failure occurs in 5–10% of cases, necessitating alternative interventions [10].

The Sengstaken–Blakemore tube (SBT) serves as a rescue therapy for life-threatening upper gastrointestinal bleeding when endoscopic treatment fails or is unavailable [11]. The device consists of three lumens and two balloons: a gastric balloon designed to anchor the tube and apply pressure at the gastroesophageal junction, and an esophageal balloon for direct compression of esophageal varices or bleeding sites [12]. While primarily indicated for variceal bleeding, SBT has been successfully used in various non-variceal bleeding scenarios [13].

Prior esophageal or gastric surgery represents a relative contraindication for SBT insertion due to altered anatomy and increased risk of perforation at surgical anastomoses [14,15]. Despite these concerns, there are limited reports describing the use of SBT in patients with previous upper gastrointestinal surgery. Here, we report a case of successful hemostasis using selective esophageal balloon inflation of the SBT in a patient with prior esophageal cancer surgery, demonstrating that judicious use of this device may be lifesaving even in the presence of relative contraindications.

## 2. Case Presentation

**Figure 1:**
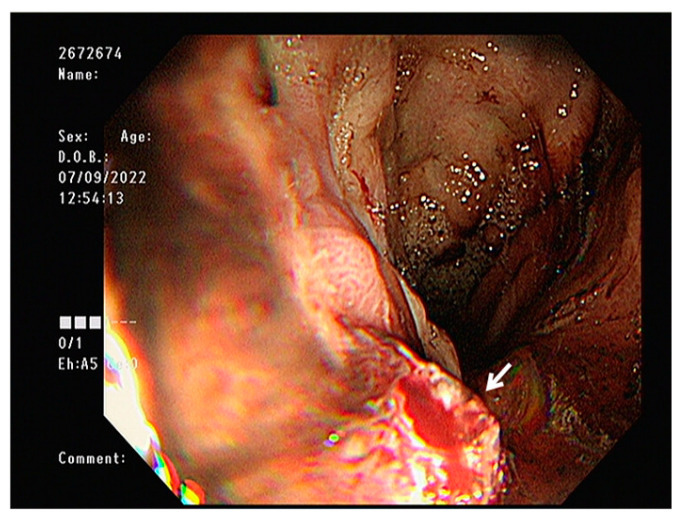
Endoscopic image showing a shallow ulcer with active bleeding located in the intrathoracic esophagus (white arrow).

**Figure 2:**
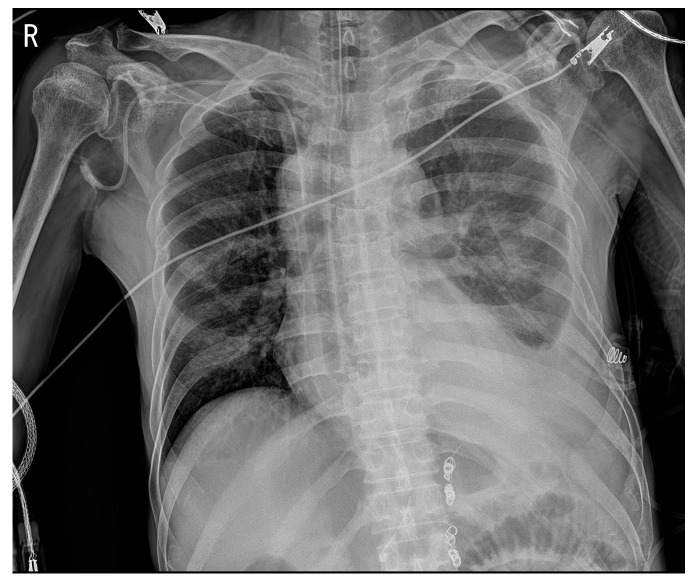
Anteroposterior chest X-ray after Sengstaken–Blakemore tube insertion showing the esophageal balloon placement at 24 cm depth.

**Figure 3:**
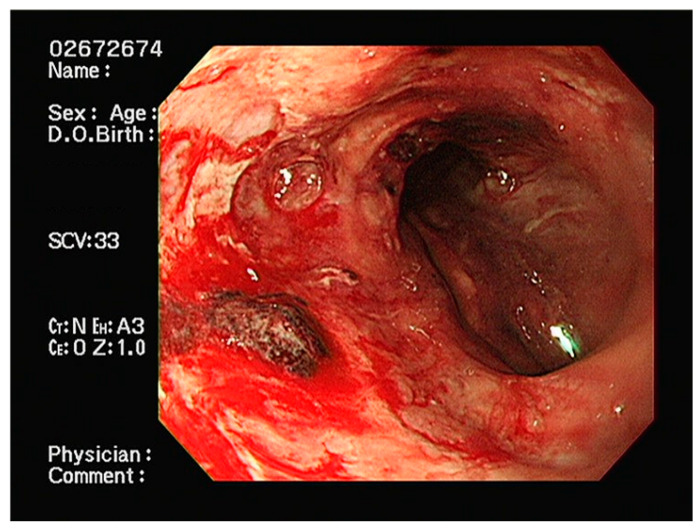
Follow-up endoscopic view after hemostasis, revealing the previously bleeding ulcer site without active hemorrhage.

A 63-year-old man presented to the emergency department with hematemesis and loss of consciousness. The patient had a history of partial splenic artery embolization with retrograde transvenous obliteration (PARTO) for alcoholic cirrhosis and gastric varices and Ivor Lewis surgery for esophageal cancer. Three years prior to presentation, an incidental abdominal computed tomography scan revealed gastric fundal and esophageal varices with gastrorenal shunt, leading to the diagnosis of alcoholic liver cirrhosis. He subsequently underwent PARTO for management of the varices. Two years prior to presentation, during follow-up surveillance endoscopy, a mid-esophageal cancer was discovered. He underwent Ivor Lewis esophagectomy for the esophageal cancer. The surgical technique involved division of the upper thoracic esophagus with esophagogastric anastomosis constructed at the level of the upper thoracic esophagus, approximately 21 cm from the incisors. The gastric conduit was created through the posterior mediastinal route. The pathology revealed moderately differentiated squamous cell carcinoma staged as pT1bN1M0. Final pathological examination confirmed clear resection margins (R0), with all harvested lymph nodes negative for tumor involvement. On arrival, the vital signs were: blood pressure 108/74 mmHg, heart rate 117 beats/min, respiratory rate 20 beats/min, temperature 36.6 °C, and the patient was lethargic. Arterial blood gas analysis showed metabolic acidosis with elevated lactic acid: pH 7.449, PaO_2_ 75.5 mmHg, PaCO_2_ 18.5 mmHg, oxygen saturation 96%, lactic acid 6.3 mmol/L, and bicarbonate 12.9 mmol/L. Complete blood count showed leukocytosis (white blood cells 13,510/µL), severe anemia (hemoglobin 6.2 g/dL), normal platelet count (177 × 10^3^/µL), and elevated serum ammonia (197.7 µg/dL; reference range: 18.7–86.9 µg/dL). Endoscopy revealed a clot-covered lesion in the intrathoracic esophagus (24 cm from front teeth), which bled from a shallow ulcer after clot removal (Figure 1). The anastomosis from Ivor Lewis surgery was located at 21 cm. Although gastric variceal bleeding and ulcers around the anastomosis site were considered, the heavy esophageal bleeding obscured clear differentiation. Band ligation was attempted at the suspected lesion but failed, as the band detached repeatedly. The patient’s condition worsened: consciousness declined, hemoglobin dropped to 5.4 g/dL, and lactic acid rose to 9.8 mmol/L. Mechanical ventilation was initiated, and an SBT was inserted (Figure 2). Because the source (ulcer vs. varices) was unclear, the SBT was used for compression at a fixed depth of 24 cm. Owing to the previous Ivor Lewis surgery, there was no gastroesophageal junction; hence, the gastric balloon and traction were omitted. Band ligation was attempted at the suspected lesion, The esophageal balloon alone was applied, and its pressure was maintained at a maximum of 40 mmHg. During inhalation, pressure transiently rose to 44 mmHg owing to positive intrathoracic pressure but normalized during exhalation, with an average maximum pressure of 40 mmHg. One 5 min decompression was performed in the emergency room. Follow-up endoscopy in the intensive care unit (ICU) revealed no active bleeding; therefore, the ulcer seen initially was confirmed as the source (Figure 3). No findings indicated variceal bleeding or ulcers around the anastomosis site, and no complications from SBT such as esophageal ischemia were observed. After applying a hemostatic agent to the ulcer area, the patient was monitored in the ICU and discharged on hospital day 20 without recurrent hematemesis.

## 3. Discussion

This case highlights the complex management challenges encountered when treating upper gastrointestinal bleeding in patients with altered anatomy following esophageal surgery. The Ivor Lewis esophagectomy fundamentally changes the normal anatomical relationships, creating a gastric conduit with an intrathoracic anastomosis that complicates both endoscopic visualization and therapeutic interventions [16,17]. The tangential approach required to access bleeding sites near the anastomosis, combined with the limited flexibility of the reconstructed anatomy, significantly reduces the success rate of standard endoscopic therapies. The development of ulceration at or near esophagogastric anastomoses occurs in 5–15% of patients following esophagectomy, with multiple contributing factors including ischemia, bile reflux, and mechanical stress at the anastomotic site [18]. In our patients, the presence of underlying cirrhosis likely contributed additional risk factors including coagulopathy, thrombocytopenia, and portal hypertension, creating a particularly challenging clinical scenario [19].

In our case, the differential diagnosis between variceal bleeding and ulcer hemorrhage was challenging due to the patient’s complex medical history. The presence of known esophageal varices from cirrhosis, combined with the post-surgical anatomy, made endoscopic visualization difficult. The heavy bleeding obscured the visual field, and the altered anatomy from the Ivor Lewis operation created tangential viewing angles that prevented definitive identification of the bleeding source initially. The patient’s previous Ivor Lewis operation significantly impacted bleeding management by eliminating the normal gastroesophageal junction, precluding standard SBT techniques, and creating a tubular gastric conduit vulnerable to perforation.

In our case, standard endoscopic modalities failed despite multiple attempts. Injection therapy with epinephrine achieved only temporary hemostasis with immediate rebleeding. Band ligation, highly effective for variceal bleeding, failed due to the shallow ulcer morphology and inability to adequately suction the lesion into the banding chamber. Hemoclips could not be deployed due to poor visualization and the tangential approach angle inherent to the tubular esophagus with altered anatomy. These technical failures underscore the limitations of conventional endoscopic techniques in post-surgical anatomy.

Recent advances such as over-the-scope clips can capture larger tissue volumes with stronger compression force, achieving success rates of 85–95% in refractory bleeding [20]. Hemostatic powders like Hemospray and EndoClot provide non-contact hemostasis through mechanical barrier formation, with immediate success rates of 80–90%, though rebleeding within 72 h necessitates definitive therapy [21,22]. However, these technologies were not available at our institution during this emergency.

Alternative approaches when endoscopy fails include transcatheter arterial embolization, with success rate of 70–90% for upper gastrointestinal bleeding [23]. However, post-esophagectomy vascular anatomy alterations, with the gastric conduit supplied primarily by right gastroepiploic and right gastric arteries, increase the conduit ischemia risk [24]. Surgical intervention carries prohibitive risk, with re-operative surgery within the first year after esophagectomy associated with mortality rates exceeding 20% [25]. Covered self-expanding metal stents offer an alternative to balloon tamponade, though migration rates of 20–30% and anastomotic perforation risk limit their utility in post-surgical anatomy [26].

When endoscopic hemostasis fails, the SBT can serve as a temporizing measure for variceal bleeding [27,28]. Standard SBT use begins with inflation of the gastric balloon with 50 mL of air to confirm stomach placement, followed by up to 200 mL and traction using a 1 L fluid bag [12]. Esophageal bleeding is typically managed by traction at the gastroesophageal junction, and the esophageal balloon is not usually inflated unless bleeding persists. if needed, the esophageal balloon is inflated to 40 mmHg and decompressed at 1–2 h intervals [13]. Airway protection with intubation is essential prior to SBT insertion [12]. Positive pressure ventilation causes esophageal balloon pressure fluctuations; pressure up to 45 mmHg may occur. Continuous monitoring is needed to avoid esophageal injury [12,13].

The mechanism of hemostasis achieved by the SBT involves direct mechanical compression of bleeding vessels, resulting in reduced venous blood flow and promoting thrombus formation at the bleeding site [29]. The balloon creates circumferential pressure that collapses the esophageal lumen and compresses submucosal vessels, effectively tamponading the hemorrhage. However, this technique carries significant risks including esophageal perforation (0.5–3%), aspiration pneumonia (10–20%), pressure necrosis, and balloon migration, with overall complication rates reported between 10 and 40% [30]. The management of upper gastrointestinal bleeding in cirrhotic patients follows fundamental principles: hemodynamic stabilization with crystalloids and blood products, correction of coagulopathy with fresh frozen plasma and vitamin, prophylactic antibiotics to prevent spontaneous bacterial peritonitis, and vasoactive drugs such as octreotide or terlipressin to reduce portal pressure [31,32]. In our case, laboratory findings including lactate elevation from 6.3 to 9.8 mmol/L, severe metabolic acidosis with pH 7.32, progressive anemia despite transfusion, and elevated base deficit indicated tissue hypoperfusion and hypovolemic shock, necessitating aggressive resuscitation and immediate intervention.

In our patient with post-Ivor Lewis anatomy, standard SBT technique required significant modification. The absence of a normal gastroesophageal junction precluded gastric balloon use and traction application. Our approach utilizing only the esophageal balloon positioned directly at the bleeding site achieved effective hemostasis while minimizing conduit rupture risk. Maintaining balloon pressure at 40 mmHg with continuous monitoring was crucial for balancing hemostatic efficacy against pressure necrosis risk. The SBT provided successful bridge therapy, with rebleeding rates of 50–60% after balloon deflation well documented in the literature, [33] emphasizing the need for definitive treatment planning.

The decision to use SBT despite the relative contraindication required careful risk-benefit analysis. Our modifications––avoiding gastric balloon inflation, maintaining limited pressure, implementing brief decompression periods, and continuous monitoring––successfully minimized complications. The literature review reveals only limited reports of SBT use in post-esophagectomy patients, with most involving variceal rather than anastomotic ulcer bleeding [34]. Our case adds important evidence that modified SBT techniques can be lifesaving when judiciously applied.

The clinically distinctive aspect of this case was the successful application of a relatively contraindicated intervention through careful modification of standard technique. This demonstrated that in life-threatening situations with limited options, creative adaptation of existing tools can be lifesaving. Post-esophagectomy anatomy requires innovative approaches, with the treating team prepared to modify conventional techniques. Timing was critical––rapid escalation from failed endoscopy to SBT insertion likely prevented irreversible deterioration. Finally, this experience demonstrated that historical techniques like the SBT retain value in modern practice when thoughtfully adapted to challenging anatomical situations, particularly when newer technologies are unavailable or inappropriate.

## 4. Conclusions

This case illustrates successful hemostasis achieved through selective esophageal ballooning of the SB tube in a patient with prior esophageal surgery—a relative contraindication. However, in emergent situations where endoscopic hemostasis fails and clinical deterioration occurs, such interventions may be warranted. Careful application of the esophageal balloon alone, with real-time pressure monitoring and avoidance of traction, enabled safe and effective bleeding control without complications. This case highlights that, with prudent clinical judgment and meticulous monitoring, even relatively contraindicated interventions may offer life-saving benefits.

## Data Availability

The original data presented in this study are available on reasonable request from the corresponding author. The data are not publicly available due to privacy concerns.
